# Bis(pyridine-κ*N*){*N* ^2^,*N* ^2′^-[1,1′-(pyridine-2,6-di­yl)diethyl­idyne]benzene­sulfono­hydrazonato-κ^5^ *O*,*N*,*N*′,*N*′′,*O*′}nickel(II)

**DOI:** 10.1107/S1600536809055639

**Published:** 2010-01-09

**Authors:** Juahir Yusnita, Hapipah Mohd Ali, Mahmood A. Abdulla, Ward T. Robinson, Hamid Khaledi

**Affiliations:** a Department of Chemistry, University of Malaya, 50603 Kuala Lumpur, Malaysia; b Department of Molecular Medicine, University of Malaya, 50603 Kuala Lumpur, Malaysia

## Abstract

In the crystal structure of the title compound, [Ni(C_21_H_19_N_5_O_4_S_2_)(C_5_H_5_N)_2_], the metal center is seven-coordinate, with an approximate penta­gonal-bipyramidal configuration. The Ni atom is chelated by a dianionic penta­dentate Schiff base *via* the pyridine N atom, the two azomethine N atoms and the two sulfonyl O atoms. The latter coordinate to Ni at different distances, *viz*. 2.3337 (12) and 2.7988 (12) Å. Two apically coordinated pyridine mol­ecules complete the seven-coordin­ate geometry. The dihedral angle between the two pyridine ring planes is 68.25 (6)°.

## Related literature

For the structure of the ligand and its zinc(II) complex, see: Yusnita *et al.* (2009*a* ▶). For the structure of copper(II) complex of a similar ligand, see: Yusnita *et al.* (2009*b* ▶).
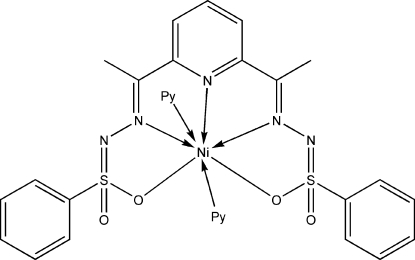


## Experimental

### 

#### Crystal data

[Ni(C_21_H_19_N_5_O_4_S_2_)(C_5_H_5_N)_2_]
*M* *_r_* = 686.44
Monoclinic, 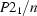

*a* = 11.6029 (2) Å
*b* = 15.8298 (3) Å
*c* = 16.4156 (3) Å
β = 91.823 (2)°
*V* = 3013.55 (9) Å^3^
*Z* = 4
Mo *K*α radiation
μ = 0.83 mm^−1^
*T* = 100 K
0.30 × 0.22 × 0.19 mm

#### Data collection

Bruker APEXII CCD diffractometer
Absorption correction: multi-scan (*SADABS*; Sheldrick, 1996 ▶) *T*_min_ = 0.788, *T*_max_ = 0.858
22997 measured reflections
5308 independent reflections
4815 reflections with *I* > 2σ(*I*)
*R*_int_ = 0.020

#### Refinement

*R*[*F* ^2^ > 2σ(*F* ^2^)] = 0.024
*wR*(*F* ^2^) = 0.065
*S* = 1.05
5308 reflections
406 parameters
H-atom parameters constrained
Δρ_max_ = 0.34 e Å^−3^
Δρ_min_ = −0.36 e Å^−3^

### 

Data collection: *APEX2* (Bruker, 2007 ▶); cell refinement: *SAINT* (Bruker, 2007 ▶); data reduction: *SAINT* program(s) used to solve structure: *SHELXS97* (Sheldrick, 2008 ▶); program(s) used to refine structure: *SHELXL97* (Sheldrick, 2008 ▶); molecular graphics: *X-SEED* (Barbour, 2001 ▶); software used to prepare material for publication: *publCIF* (Westrip, 2010 ▶).

## Supplementary Material

Crystal structure: contains datablocks I, global. DOI: 10.1107/S1600536809055639/om2309sup1.cif

Structure factors: contains datablocks I. DOI: 10.1107/S1600536809055639/om2309Isup2.hkl

Additional supplementary materials:  crystallographic information; 3D view; checkCIF report

## Figures and Tables

**Fig. 1.**
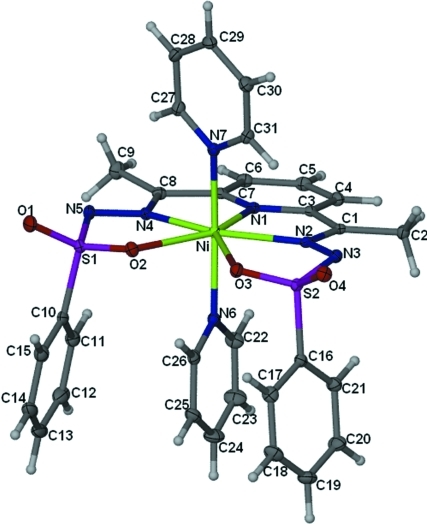
Thermal ellipsoid plot of the title compound at the 40% probability level. Hydrogen atoms are drawn as spheres of arbitrary radius.

## supplementary crystallographic information

### Experimental

2,6-Diacetylpyridinebis(benzenesulfonylhydrazide) (1.413 g, 3 mmol) was dissolved in ethanol (50 ml) and three droplets of triethylamine were added, followed by addition of an ethanolic solution of stoichiometric amount of hydrated nickel (II) acetate. The mixture was refluxed for 5 h. The resulting dark brown solids were filtered and dried over silica gel. Brown crystals of the title compound were grown by slow evaporation of a pyridine solution.

### Refinement

Hydrogen atoms were placed at calculated positions (C–H 0.95–0.98 Å), and were treated as riding on their parent atoms, with *U*(H) set to 1.2–1.5 times *U*eq(C).

### Crystal data

**Table:**

[Ni(C_21_H_19_N_5_O_4_S_2_)(C_5_H_5_N)_2_]	*F*(000) = 1428
*M**_r_* = 686.44	*D*_x_ = 1.515 Mg m^−^^3^
Monoclinic, *P*2_1_/*n*	Mo *K*α radiation, λ = 0.71073 Å
Hall symbol: -P 2yn	Cell parameters from 9879 reflections
*a* = 11.6029 (2) Å	θ = 2.5–28.3°
*b* = 15.8298 (3) Å	µ = 0.83 mm^−^^1^
*c* = 16.4156 (3) Å	*T* = 100 K
β = 91.823 (2)°	Block, brown
*V* = 3013.55 (9) Å^3^	0.30 × 0.22 × 0.19 mm
*Z* = 4	

### Data collection

**Table:**

Bruker APEXII CCD diffractometer	5308 independent reflections
Radiation source: fine-focus sealed tube	4815 reflections with *I* > 2σ(*I*)
graphite	*R*_int_ = 0.020
φ and ω scans	θ_max_ = 25.0°, θ_min_ = 1.8°
Absorption correction: multi-scan (*SADABS*; Sheldrick, 1996)	*h* = −13→13
*T*_min_ = 0.788, *T*_max_ = 0.858	*k* = −18→18
22997 measured reflections	*l* = −19→19

### Refinement

**Table:**

Refinement on *F*^2^	Primary atom site location: structure-invariant direct methods
Least-squares matrix: full	Secondary atom site location: difference Fourier map
*R*[*F*^2^ > 2σ(*F*^2^)] = 0.024	Hydrogen site location: inferred from neighbouring sites
*wR*(*F*^2^) = 0.065	H-atom parameters constrained
*S* = 1.05	*w* = 1/[σ^2^(*F*_o_^2^) + (0.0291*P*)^2^ + 2.4638*P*] where *P* = (*F*_o_^2^ + 2*F*_c_^2^)/3
5308 reflections	(Δ/σ)_max_ = 0.001
406 parameters	Δρ_max_ = 0.34 e Å^−^^3^
0 restraints	Δρ_min_ = −0.36 e Å^−^^3^

### Special details

**Table:**

Geometry. All e.s.d.'s (except the e.s.d. in the dihedral angle between two l.s. planes) are estimated using the full covariance matrix. The cell e.s.d.'s are taken into account individually in the estimation of e.s.d.'s in distances, angles and torsion angles; correlations between e.s.d.'s in cell parameters are only used when they are defined by crystal symmetry. An approximate (isotropic) treatment of cell e.s.d.'s is used for estimating e.s.d.'s involving l.s. planes.
Refinement. Refinement of *F*^2^ against ALL reflections. The weighted *R*-factor *wR* and goodness of fit *S* are based on *F*^2^, conventional *R*-factors *R* are based on *F*, with *F* set to zero for negative *F*^2^. The threshold expression of *F*^2^ > σ(*F*^2^) is used only for calculating *R*-factors(gt) *etc*. and is not relevant to the choice of reflections for refinement. *R*-factors based on *F*^2^ are statistically about twice as large as those based on *F*, and *R*- factors based on ALL data will be even larger.

### Fractional atomic coordinates and isotropic or equivalent isotropic displacement parameters (Å^2^)

**Table:**

	*x*	*y*	*z*	*U*_iso_*/*U*_eq_	
Ni	0.410517 (18)	0.141485 (13)	0.215880 (12)	0.01157 (7)	
S1	0.59292 (4)	−0.00687 (3)	0.18463 (3)	0.01400 (10)	
S2	0.27045 (4)	0.12194 (3)	0.40344 (2)	0.01272 (10)	
O1	0.60699 (11)	−0.09339 (8)	0.16025 (8)	0.0207 (3)	
O2	0.49868 (10)	0.01082 (7)	0.23919 (7)	0.0157 (3)	
O3	0.33543 (10)	0.06141 (7)	0.35665 (7)	0.0166 (3)	
O4	0.17478 (10)	0.08872 (8)	0.44772 (7)	0.0174 (3)	
N1	0.38858 (12)	0.24587 (9)	0.14898 (8)	0.0127 (3)	
N2	0.28461 (12)	0.21687 (9)	0.28339 (8)	0.0125 (3)	
N3	0.22201 (12)	0.19994 (9)	0.35170 (8)	0.0137 (3)	
N4	0.52548 (12)	0.12310 (9)	0.12007 (9)	0.0134 (3)	
N5	0.58722 (12)	0.05107 (9)	0.10487 (9)	0.0156 (3)	
N6	0.53749 (12)	0.19065 (9)	0.29575 (9)	0.0143 (3)	
N7	0.27701 (12)	0.07348 (9)	0.15452 (9)	0.0140 (3)	
C1	0.25986 (14)	0.28945 (11)	0.24972 (10)	0.0131 (3)	
C2	0.17637 (15)	0.35009 (11)	0.28509 (11)	0.0169 (4)	
H2A	0.1036	0.3209	0.2948	0.025*	
H2B	0.1623	0.3968	0.2469	0.025*	
H2C	0.2084	0.3723	0.3367	0.025*	
C3	0.32043 (14)	0.30847 (11)	0.17453 (10)	0.0131 (3)	
C4	0.31328 (15)	0.38448 (11)	0.13167 (11)	0.0166 (4)	
H4	0.2653	0.4290	0.1494	0.020*	
C5	0.37776 (15)	0.39357 (11)	0.06257 (11)	0.0182 (4)	
H5	0.3742	0.4450	0.0328	0.022*	
C6	0.44751 (15)	0.32834 (11)	0.03648 (10)	0.0163 (4)	
H6	0.4913	0.3342	−0.0111	0.020*	
C7	0.45153 (14)	0.25424 (11)	0.08200 (10)	0.0139 (3)	
C8	0.52355 (14)	0.18075 (11)	0.06381 (10)	0.0145 (4)	
C9	0.58566 (16)	0.17241 (12)	−0.01367 (11)	0.0209 (4)	
H9A	0.5851	0.2268	−0.0421	0.031*	
H9B	0.5473	0.1297	−0.0483	0.031*	
H9C	0.6655	0.1551	−0.0017	0.031*	
C10	0.72211 (15)	0.02117 (11)	0.24006 (11)	0.0169 (4)	
C11	0.78134 (15)	0.09477 (12)	0.22277 (11)	0.0208 (4)	
H11	0.7556	0.1304	0.1793	0.025*	
C12	0.87894 (17)	0.11586 (14)	0.26986 (12)	0.0275 (5)	
H12	0.9197	0.1665	0.2590	0.033*	
C13	0.91686 (16)	0.06321 (15)	0.33268 (12)	0.0305 (5)	
H13	0.9841	0.0775	0.3642	0.037*	
C14	0.85730 (17)	−0.00988 (14)	0.34959 (12)	0.0288 (5)	
H14	0.8834	−0.0455	0.3930	0.035*	
C15	0.75943 (16)	−0.03152 (13)	0.30345 (11)	0.0227 (4)	
H15	0.7183	−0.0818	0.3150	0.027*	
C16	0.36809 (14)	0.16219 (11)	0.48003 (10)	0.0140 (3)	
C17	0.41986 (15)	0.10464 (12)	0.53378 (11)	0.0192 (4)	
H17	0.4048	0.0459	0.5280	0.023*	
C18	0.49334 (17)	0.13345 (13)	0.59570 (11)	0.0233 (4)	
H18	0.5288	0.0945	0.6328	0.028*	
C19	0.51522 (16)	0.21932 (13)	0.60359 (11)	0.0236 (4)	
H19	0.5656	0.2392	0.6461	0.028*	
C20	0.46384 (17)	0.27609 (12)	0.54965 (12)	0.0235 (4)	
H20	0.4792	0.3348	0.5553	0.028*	
C21	0.39001 (15)	0.24778 (11)	0.48730 (11)	0.0188 (4)	
H21	0.3550	0.2867	0.4501	0.023*	
C22	0.57825 (16)	0.26962 (12)	0.28800 (12)	0.0211 (4)	
H22	0.5490	0.3033	0.2442	0.025*	
C23	0.66049 (17)	0.30403 (13)	0.34058 (13)	0.0277 (5)	
H23	0.6867	0.3603	0.3331	0.033*	
C24	0.70418 (17)	0.25566 (13)	0.40424 (12)	0.0267 (4)	
H24	0.7616	0.2776	0.4409	0.032*	
C25	0.66268 (15)	0.17464 (12)	0.41350 (11)	0.0205 (4)	
H25	0.6906	0.1400	0.4571	0.025*	
C26	0.57989 (15)	0.14469 (11)	0.35850 (10)	0.0161 (4)	
H26	0.5516	0.0889	0.3654	0.019*	
C27	0.28759 (15)	0.04331 (11)	0.07836 (11)	0.0170 (4)	
H27	0.3566	0.0548	0.0508	0.020*	
C28	0.20283 (16)	−0.00357 (11)	0.03843 (11)	0.0197 (4)	
H28	0.2130	−0.0225	−0.0158	0.024*	
C29	0.10303 (16)	−0.02267 (12)	0.07827 (11)	0.0203 (4)	
H29	0.0444	−0.0561	0.0526	0.024*	
C30	0.09044 (16)	0.00798 (12)	0.15641 (12)	0.0205 (4)	
H30	0.0227	−0.0039	0.1854	0.025*	
C31	0.17797 (15)	0.05617 (11)	0.19157 (11)	0.0178 (4)	
H31	0.1677	0.0782	0.2447	0.021*	

### Atomic displacement parameters (Å^2^)

**Table:**

	*U*^11^	*U*^22^	*U*^33^	*U*^12^	*U*^13^	*U*^23^
Ni	0.01215 (12)	0.01122 (12)	0.01132 (12)	0.00032 (8)	0.00009 (8)	0.00054 (8)
S1	0.0129 (2)	0.0136 (2)	0.0155 (2)	0.00219 (16)	0.00011 (16)	−0.00047 (16)
S2	0.0127 (2)	0.0124 (2)	0.0131 (2)	−0.00030 (16)	0.00112 (16)	−0.00026 (16)
O1	0.0209 (7)	0.0145 (6)	0.0266 (7)	0.0039 (5)	−0.0017 (5)	−0.0031 (5)
O2	0.0139 (6)	0.0156 (6)	0.0177 (6)	0.0009 (5)	0.0019 (5)	0.0016 (5)
O3	0.0159 (6)	0.0149 (6)	0.0191 (6)	0.0006 (5)	0.0013 (5)	−0.0028 (5)
O4	0.0165 (6)	0.0180 (6)	0.0180 (6)	−0.0024 (5)	0.0031 (5)	0.0015 (5)
N1	0.0128 (7)	0.0137 (7)	0.0113 (7)	0.0001 (6)	−0.0011 (6)	−0.0001 (6)
N2	0.0125 (7)	0.0146 (7)	0.0104 (7)	−0.0016 (6)	−0.0004 (5)	−0.0016 (6)
N3	0.0140 (7)	0.0162 (7)	0.0110 (7)	0.0011 (6)	0.0018 (6)	−0.0008 (6)
N4	0.0117 (7)	0.0145 (7)	0.0140 (7)	0.0014 (6)	0.0000 (6)	−0.0017 (6)
N5	0.0153 (7)	0.0167 (8)	0.0149 (7)	0.0040 (6)	0.0022 (6)	−0.0016 (6)
N6	0.0126 (7)	0.0154 (7)	0.0150 (7)	0.0002 (6)	0.0025 (6)	−0.0004 (6)
N7	0.0150 (7)	0.0122 (7)	0.0147 (7)	0.0027 (6)	−0.0009 (6)	−0.0003 (6)
C1	0.0129 (8)	0.0146 (8)	0.0116 (8)	0.0003 (7)	−0.0024 (6)	−0.0024 (7)
C2	0.0184 (9)	0.0177 (9)	0.0146 (9)	0.0042 (7)	0.0006 (7)	−0.0011 (7)
C3	0.0117 (8)	0.0134 (8)	0.0140 (8)	−0.0004 (7)	−0.0029 (7)	−0.0022 (7)
C4	0.0180 (9)	0.0134 (9)	0.0181 (9)	0.0013 (7)	−0.0025 (7)	0.0001 (7)
C5	0.0211 (9)	0.0150 (9)	0.0183 (9)	−0.0024 (7)	−0.0037 (7)	0.0042 (7)
C6	0.0171 (9)	0.0177 (9)	0.0138 (9)	−0.0024 (7)	−0.0005 (7)	0.0029 (7)
C7	0.0121 (8)	0.0169 (9)	0.0126 (8)	−0.0023 (7)	−0.0018 (7)	−0.0004 (7)
C8	0.0133 (8)	0.0167 (9)	0.0136 (9)	−0.0022 (7)	0.0002 (7)	0.0007 (7)
C9	0.0228 (10)	0.0223 (10)	0.0179 (9)	0.0012 (8)	0.0058 (8)	0.0013 (8)
C10	0.0131 (8)	0.0220 (9)	0.0156 (9)	0.0043 (7)	0.0018 (7)	−0.0046 (7)
C11	0.0159 (9)	0.0259 (10)	0.0207 (10)	0.0005 (8)	0.0034 (7)	−0.0033 (8)
C12	0.0169 (9)	0.0357 (12)	0.0301 (11)	−0.0047 (8)	0.0054 (8)	−0.0115 (9)
C13	0.0138 (9)	0.0548 (14)	0.0226 (10)	0.0055 (9)	−0.0012 (8)	−0.0169 (10)
C14	0.0240 (10)	0.0454 (13)	0.0168 (10)	0.0133 (10)	−0.0029 (8)	−0.0057 (9)
C15	0.0223 (10)	0.0274 (10)	0.0184 (10)	0.0060 (8)	0.0023 (8)	−0.0016 (8)
C16	0.0124 (8)	0.0177 (9)	0.0120 (8)	0.0006 (7)	0.0021 (6)	−0.0001 (7)
C17	0.0200 (9)	0.0176 (9)	0.0201 (10)	−0.0009 (7)	0.0014 (7)	0.0039 (7)
C18	0.0224 (10)	0.0284 (11)	0.0188 (10)	0.0011 (8)	−0.0038 (8)	0.0077 (8)
C19	0.0223 (10)	0.0307 (11)	0.0173 (9)	−0.0025 (8)	−0.0058 (8)	−0.0014 (8)
C20	0.0268 (10)	0.0180 (9)	0.0254 (10)	−0.0020 (8)	−0.0055 (8)	−0.0034 (8)
C21	0.0202 (9)	0.0170 (9)	0.0191 (9)	0.0018 (7)	−0.0028 (7)	0.0012 (7)
C22	0.0206 (9)	0.0197 (10)	0.0228 (10)	−0.0045 (8)	−0.0012 (8)	0.0045 (8)
C23	0.0260 (10)	0.0245 (10)	0.0324 (11)	−0.0115 (8)	−0.0042 (9)	0.0029 (9)
C24	0.0200 (10)	0.0345 (12)	0.0251 (11)	−0.0067 (9)	−0.0054 (8)	−0.0033 (9)
C25	0.0161 (9)	0.0290 (10)	0.0164 (9)	0.0024 (8)	−0.0012 (7)	0.0020 (8)
C26	0.0144 (9)	0.0183 (9)	0.0158 (9)	0.0021 (7)	0.0038 (7)	0.0000 (7)
C27	0.0188 (9)	0.0163 (9)	0.0160 (9)	0.0020 (7)	0.0019 (7)	−0.0006 (7)
C28	0.0234 (10)	0.0200 (9)	0.0156 (9)	0.0048 (8)	−0.0018 (7)	−0.0053 (7)
C29	0.0180 (9)	0.0174 (9)	0.0249 (10)	0.0018 (7)	−0.0062 (8)	−0.0059 (8)
C30	0.0152 (9)	0.0235 (10)	0.0227 (10)	0.0003 (8)	0.0012 (7)	−0.0034 (8)
C31	0.0184 (9)	0.0189 (9)	0.0163 (9)	0.0018 (7)	0.0015 (7)	−0.0036 (7)

### Geometric parameters (Å, °)

**Table:**

Ni—N1	1.9959 (14)	C9—H9C	0.9800
Ni—N6	2.0910 (14)	C10—C11	1.387 (3)
Ni—N4	2.1145 (14)	C10—C15	1.392 (3)
Ni—N7	2.1152 (14)	C11—C12	1.391 (3)
Ni—N2	2.2111 (14)	C11—H11	0.9500
Ni—O2	2.3337 (12)	C12—C13	1.387 (3)
Ni—O3	2.7988 (12)	C12—H12	0.9500
S1—O1	1.4376 (13)	C13—C14	1.380 (3)
S1—O2	1.4624 (12)	C13—H13	0.9500
S1—N5	1.5984 (15)	C14—C15	1.388 (3)
S1—C10	1.7847 (18)	C14—H14	0.9500
S2—O4	1.4450 (12)	C15—H15	0.9500
S2—O3	1.4538 (12)	C16—C21	1.383 (3)
S2—N3	1.5911 (15)	C16—C17	1.391 (2)
S2—C16	1.7832 (17)	C17—C18	1.383 (3)
N1—C3	1.343 (2)	C17—H17	0.9500
N1—C7	1.346 (2)	C18—C19	1.388 (3)
N2—C1	1.303 (2)	C18—H18	0.9500
N2—N3	1.3816 (19)	C19—C20	1.383 (3)
N4—C8	1.298 (2)	C19—H19	0.9500
N4—N5	1.374 (2)	C20—C21	1.388 (3)
N6—C26	1.342 (2)	C20—H20	0.9500
N6—C22	1.344 (2)	C21—H21	0.9500
N7—C31	1.345 (2)	C22—C23	1.378 (3)
N7—C27	1.348 (2)	C22—H22	0.9500
C1—C3	1.471 (2)	C23—C24	1.379 (3)
C1—C2	1.494 (2)	C23—H23	0.9500
C2—H2A	0.9800	C24—C25	1.380 (3)
C2—H2B	0.9800	C24—H24	0.9500
C2—H2C	0.9800	C25—C26	1.381 (3)
C3—C4	1.395 (2)	C25—H25	0.9500
C4—C5	1.386 (3)	C26—H26	0.9500
C4—H4	0.9500	C27—C28	1.381 (3)
C5—C6	1.388 (3)	C27—H27	0.9500
C5—H5	0.9500	C28—C29	1.381 (3)
C6—C7	1.391 (2)	C28—H28	0.9500
C6—H6	0.9500	C29—C30	1.384 (3)
C7—C8	1.469 (2)	C29—H29	0.9500
C8—C9	1.488 (2)	C30—C31	1.382 (3)
C9—H9A	0.9800	C30—H30	0.9500
C9—H9B	0.9800	C31—H31	0.9500
			
N1—Ni—N6	96.42 (6)	N4—C8—C7	114.16 (15)
N1—Ni—N4	77.12 (6)	N4—C8—C9	123.23 (16)
N6—Ni—N4	93.96 (5)	C7—C8—C9	122.58 (15)
N1—Ni—N7	94.63 (6)	C8—C9—H9A	109.5
N6—Ni—N7	168.17 (6)	C8—C9—H9B	109.5
N4—Ni—N7	92.61 (5)	H9A—C9—H9B	109.5
N1—Ni—N2	75.77 (5)	C8—C9—H9C	109.5
N6—Ni—N2	87.04 (5)	H9A—C9—H9C	109.5
N4—Ni—N2	152.81 (5)	H9B—C9—H9C	109.5
N7—Ni—N2	91.59 (5)	C11—C10—C15	120.81 (17)
N1—Ni—O2	150.64 (5)	C11—C10—S1	121.21 (14)
N6—Ni—O2	86.02 (5)	C15—C10—S1	117.93 (14)
N4—Ni—O2	73.52 (5)	C10—C11—C12	119.19 (18)
N7—Ni—O2	86.42 (5)	C10—C11—H11	120.4
N2—Ni—O2	133.58 (5)	C12—C11—H11	120.4
N1—Ni—O3	142.34 (5)	C13—C12—C11	120.2 (2)
N6—Ni—O3	83.25 (5)	C13—C12—H12	119.9
N4—Ni—O3	140.54 (5)	C11—C12—H12	119.9
N7—Ni—O3	85.42 (5)	C14—C13—C12	120.28 (18)
N2—Ni—O3	66.59 (4)	C14—C13—H13	119.9
O2—Ni—O3	67.02 (4)	C12—C13—H13	119.9
O1—S1—O2	116.53 (7)	C13—C14—C15	120.23 (19)
O1—S1—N5	108.70 (8)	C13—C14—H14	119.9
O2—S1—N5	112.31 (7)	C15—C14—H14	119.9
O1—S1—C10	106.14 (8)	C14—C15—C10	119.33 (19)
O2—S1—C10	105.81 (8)	C14—C15—H15	120.3
N5—S1—C10	106.69 (8)	C10—C15—H15	120.3
O4—S2—O3	116.46 (7)	C21—C16—C17	120.82 (16)
O4—S2—N3	106.68 (7)	C21—C16—S2	121.46 (13)
O3—S2—N3	114.18 (7)	C17—C16—S2	117.71 (14)
O4—S2—C16	104.86 (7)	C18—C17—C16	119.59 (17)
O3—S2—C16	106.23 (7)	C18—C17—H17	120.2
N3—S2—C16	107.75 (8)	C16—C17—H17	120.2
S1—O2—Ni	113.69 (7)	C17—C18—C19	119.87 (17)
S2—O3—Ni	108.55 (6)	C17—C18—H18	120.1
C3—N1—C7	121.51 (15)	C19—C18—H18	120.1
C3—N1—Ni	120.33 (11)	C20—C19—C18	120.18 (18)
C7—N1—Ni	117.91 (11)	C20—C19—H19	119.9
C1—N2—N3	113.63 (14)	C18—C19—H19	119.9
C1—N2—Ni	113.80 (11)	C19—C20—C21	120.38 (18)
N3—N2—Ni	132.44 (11)	C19—C20—H20	119.8
N2—N3—S2	113.45 (11)	C21—C20—H20	119.8
C8—N4—N5	116.85 (14)	C16—C21—C20	119.16 (17)
C8—N4—Ni	115.81 (11)	C16—C21—H21	120.4
N5—N4—Ni	126.47 (11)	C20—C21—H21	120.4
N4—N5—S1	109.62 (11)	N6—C22—C23	123.16 (17)
C26—N6—C22	117.09 (15)	N6—C22—H22	118.4
C26—N6—Ni	120.89 (12)	C23—C22—H22	118.4
C22—N6—Ni	122.00 (12)	C22—C23—C24	119.05 (18)
C31—N7—C27	116.84 (15)	C22—C23—H23	120.5
C31—N7—Ni	120.77 (12)	C24—C23—H23	120.5
C27—N7—Ni	122.36 (12)	C23—C24—C25	118.58 (18)
N2—C1—C3	115.57 (15)	C23—C24—H24	120.7
N2—C1—C2	122.58 (15)	C25—C24—H24	120.7
C3—C1—C2	121.85 (15)	C24—C25—C26	118.99 (17)
C1—C2—H2A	109.5	C24—C25—H25	120.5
C1—C2—H2B	109.5	C26—C25—H25	120.5
H2A—C2—H2B	109.5	N6—C26—C25	123.11 (17)
C1—C2—H2C	109.5	N6—C26—H26	118.4
H2A—C2—H2C	109.5	C25—C26—H26	118.4
H2B—C2—H2C	109.5	N7—C27—C28	123.12 (17)
N1—C3—C4	120.33 (15)	N7—C27—H27	118.4
N1—C3—C1	114.38 (15)	C28—C27—H27	118.4
C4—C3—C1	125.27 (15)	C27—C28—C29	119.21 (17)
C5—C4—C3	118.51 (16)	C27—C28—H28	120.4
C5—C4—H4	120.7	C29—C28—H28	120.4
C3—C4—H4	120.7	C28—C29—C30	118.49 (17)
C4—C5—C6	120.69 (16)	C28—C29—H29	120.8
C4—C5—H5	119.7	C30—C29—H29	120.8
C6—C5—H5	119.7	C31—C30—C29	118.88 (17)
C5—C6—C7	118.14 (16)	C31—C30—H30	120.6
C5—C6—H6	120.9	C29—C30—H30	120.6
C7—C6—H6	120.9	N7—C31—C30	123.42 (16)
N1—C7—C6	120.81 (16)	N7—C31—H31	118.3
N1—C7—C8	114.53 (15)	C30—C31—H31	118.3
C6—C7—C8	124.65 (15)		
			
O1—S1—O2—Ni	−145.14 (7)	O3—Ni—N7—C31	33.11 (13)
N5—S1—O2—Ni	−18.82 (10)	N1—Ni—N7—C27	72.98 (14)
C10—S1—O2—Ni	97.20 (8)	N6—Ni—N7—C27	−128.0 (3)
N1—Ni—O2—S1	8.70 (14)	N4—Ni—N7—C27	−4.30 (14)
N6—Ni—O2—S1	−87.33 (8)	N2—Ni—N7—C27	148.83 (13)
N4—Ni—O2—S1	7.99 (7)	O2—Ni—N7—C27	−77.61 (13)
N7—Ni—O2—S1	101.78 (8)	O3—Ni—N7—C27	−144.81 (13)
N2—Ni—O2—S1	−169.43 (6)	N3—N2—C1—C3	−177.23 (13)
O3—Ni—O2—S1	−171.68 (8)	Ni—N2—C1—C3	−0.80 (18)
O4—S2—O3—Ni	145.87 (6)	N3—N2—C1—C2	3.3 (2)
N3—S2—O3—Ni	20.78 (9)	Ni—N2—C1—C2	179.72 (12)
C16—S2—O3—Ni	−97.82 (7)	C7—N1—C3—C4	0.0 (2)
N1—Ni—O3—S2	−12.32 (11)	Ni—N1—C3—C4	174.16 (12)
N6—Ni—O3—S2	79.45 (7)	C7—N1—C3—C1	−178.66 (15)
N4—Ni—O3—S2	167.49 (7)	Ni—N1—C3—C1	−4.52 (19)
N7—Ni—O3—S2	−103.97 (7)	N2—C1—C3—N1	3.3 (2)
N2—Ni—O3—S2	−10.24 (6)	C2—C1—C3—N1	−177.22 (15)
O2—Ni—O3—S2	167.98 (8)	N2—C1—C3—C4	−175.31 (16)
N6—Ni—N1—C3	−82.17 (13)	C2—C1—C3—C4	4.2 (3)
N4—Ni—N1—C3	−174.78 (13)	N1—C3—C4—C5	0.0 (3)
N7—Ni—N1—C3	93.59 (13)	C1—C3—C4—C5	178.56 (16)
N2—Ni—N1—C3	3.12 (12)	C3—C4—C5—C6	0.3 (3)
O2—Ni—N1—C3	−175.48 (10)	C4—C5—C6—C7	−0.6 (3)
O3—Ni—N1—C3	5.10 (17)	C3—N1—C7—C6	−0.4 (2)
N6—Ni—N1—C7	92.18 (12)	Ni—N1—C7—C6	−174.65 (12)
N4—Ni—N1—C7	−0.43 (12)	C3—N1—C7—C8	178.48 (14)
N7—Ni—N1—C7	−92.05 (12)	Ni—N1—C7—C8	4.20 (19)
N2—Ni—N1—C7	177.47 (13)	C5—C6—C7—N1	0.6 (3)
O2—Ni—N1—C7	−1.13 (19)	C5—C6—C7—C8	−178.08 (16)
O3—Ni—N1—C7	179.45 (10)	N5—N4—C8—C7	177.13 (14)
N1—Ni—N2—C1	−1.11 (11)	Ni—N4—C8—C7	7.13 (19)
N6—Ni—N2—C1	96.28 (12)	N5—N4—C8—C9	−1.0 (2)
N4—Ni—N2—C1	3.37 (19)	Ni—N4—C8—C9	−171.04 (13)
N7—Ni—N2—C1	−95.48 (12)	N1—C7—C8—N4	−7.5 (2)
O2—Ni—N2—C1	177.94 (10)	C6—C7—C8—N4	171.34 (16)
O3—Ni—N2—C1	−179.80 (13)	N1—C7—C8—C9	170.72 (16)
N1—Ni—N2—N3	174.46 (15)	C6—C7—C8—C9	−10.5 (3)
N6—Ni—N2—N3	−88.15 (14)	O1—S1—C10—C11	130.91 (15)
N4—Ni—N2—N3	178.94 (12)	O2—S1—C10—C11	−104.67 (15)
N7—Ni—N2—N3	80.09 (14)	N5—S1—C10—C11	15.11 (17)
O2—Ni—N2—N3	−6.49 (17)	O1—S1—C10—C15	−51.50 (16)
O3—Ni—N2—N3	−4.23 (13)	O2—S1—C10—C15	72.92 (15)
C1—N2—N3—S2	−167.49 (12)	N5—S1—C10—C15	−167.30 (14)
Ni—N2—N3—S2	16.94 (18)	C15—C10—C11—C12	−0.2 (3)
O4—S2—N3—N2	−155.04 (11)	S1—C10—C11—C12	177.28 (14)
O3—S2—N3—N2	−24.92 (14)	C10—C11—C12—C13	0.7 (3)
C16—S2—N3—N2	92.82 (12)	C11—C12—C13—C14	−0.8 (3)
N1—Ni—N4—C8	−3.93 (12)	C12—C13—C14—C15	0.5 (3)
N6—Ni—N4—C8	−99.62 (13)	C13—C14—C15—C10	0.0 (3)
N7—Ni—N4—C8	90.22 (13)	C11—C10—C15—C14	−0.1 (3)
N2—Ni—N4—C8	−8.4 (2)	S1—C10—C15—C14	−177.72 (14)
O2—Ni—N4—C8	175.71 (13)	O4—S2—C16—C21	−112.94 (15)
O3—Ni—N4—C8	176.19 (10)	O3—S2—C16—C21	123.19 (15)
N1—Ni—N4—N5	−172.83 (14)	N3—S2—C16—C21	0.43 (17)
N6—Ni—N4—N5	91.48 (13)	O4—S2—C16—C17	65.61 (15)
N7—Ni—N4—N5	−78.68 (13)	O3—S2—C16—C17	−58.26 (15)
N2—Ni—N4—N5	−177.28 (11)	N3—S2—C16—C17	178.98 (13)
O2—Ni—N4—N5	6.81 (12)	C21—C16—C17—C18	0.6 (3)
O3—Ni—N4—N5	7.29 (17)	S2—C16—C17—C18	−177.96 (14)
C8—N4—N5—S1	172.91 (12)	C16—C17—C18—C19	−0.2 (3)
Ni—N4—N5—S1	−18.29 (16)	C17—C18—C19—C20	−0.1 (3)
O1—S1—N5—N4	153.45 (11)	C18—C19—C20—C21	0.1 (3)
O2—S1—N5—N4	23.01 (13)	C17—C16—C21—C20	−0.6 (3)
C10—S1—N5—N4	−92.48 (12)	S2—C16—C21—C20	177.89 (14)
N1—Ni—N6—C26	176.50 (13)	C19—C20—C21—C16	0.3 (3)
N4—Ni—N6—C26	−106.03 (13)	C26—N6—C22—C23	0.4 (3)
N7—Ni—N6—C26	17.5 (3)	Ni—N6—C22—C23	178.89 (15)
N2—Ni—N6—C26	101.19 (13)	N6—C22—C23—C24	0.3 (3)
O2—Ni—N6—C26	−32.88 (13)	C22—C23—C24—C25	−0.8 (3)
O3—Ni—N6—C26	34.42 (12)	C23—C24—C25—C26	0.5 (3)
N1—Ni—N6—C22	−1.92 (14)	C22—N6—C26—C25	−0.7 (2)
N4—Ni—N6—C22	75.54 (14)	Ni—N6—C26—C25	−179.16 (13)
N7—Ni—N6—C22	−160.9 (2)	C24—C25—C26—N6	0.2 (3)
N2—Ni—N6—C22	−77.23 (14)	C31—N7—C27—C28	−0.2 (3)
O2—Ni—N6—C22	148.70 (14)	Ni—N7—C27—C28	177.80 (13)
O3—Ni—N6—C22	−144.00 (14)	N7—C27—C28—C29	−1.6 (3)
N1—Ni—N7—C31	−109.10 (13)	C27—C28—C29—C30	1.7 (3)
N6—Ni—N7—C31	49.9 (3)	C28—C29—C30—C31	−0.2 (3)
N4—Ni—N7—C31	173.62 (13)	C27—N7—C31—C30	1.8 (3)
N2—Ni—N7—C31	−33.25 (13)	Ni—N7—C31—C30	−176.19 (14)
O2—Ni—N7—C31	100.31 (13)	C29—C30—C31—N7	−1.7 (3)
